# p53 Represses the Oncogenic Sno-MiR-28 Derived from a SnoRNA

**DOI:** 10.1371/journal.pone.0129190

**Published:** 2015-06-10

**Authors:** Feng Yu, Cameron P. Bracken, Katherine A. Pillman, David M. Lawrence, Gregory J. Goodall, David F. Callen, Paul M. Neilsen

**Affiliations:** 1 Centre for Personalized Cancer Medicine, University of Adelaide, Adelaide, SA, Australia; 2 Discipline of Medicine, University of Adelaide, Adelaide, SA, Australia; 3 Centre for Cancer Biology, SA Pathology, Adelaide, SA, Australia; 4 ACRF Cancer Genomics Facility, Centre for Cancer Biology, SA Pathology, Adelaide, Australia; 5 School of Molecular and Biomedical Science, University of Adelaide, Adelaide, Australia; 6 Swinburne University of Technology, Kuching, Sarawak, Malaysia; French National Center for Scientific Research—Institut de biologie moléculaire et cellulaire, FRANCE

## Abstract

p53 is a master tumour repressor that participates in vast regulatory networks, including feedback loops involving microRNAs (miRNAs) that regulate p53 and that themselves are direct p53 transcriptional targets. We show here that a group of polycistronic miRNA-like non-coding RNAs derived from small nucleolar RNAs (sno-miRNAs) are transcriptionally repressed by p53 through their host gene, *SNHG1*. The most abundant of these, sno-miR-28, directly targets the p53-stabilizing gene, TAF9B. Collectively, p53, SNHG1, sno-miR-28 and TAF9B form a regulatory loop which affects p53 stability and downstream p53-regulated pathways. In addition, SNHG1, SNORD28 and sno-miR-28 are all significantly upregulated in breast tumours and the overexpression of sno-miR-28 promotes breast epithelial cell proliferation. This research has broadened our knowledge of the crosstalk between small non-coding RNA pathways and roles of sno-miRNAs in p53 regulation.

## Introduction

The p53 tumour suppressor plays a pivotal role in the prevention of oncogenic transformation as highlighted by the fact that over half of all tumours have mutations in *TP53*. Cellular insults such as DNA damage or aberrant oncogene expression engage the p53 pathway, resulting in rapid stabilization of p53 protein levels. Upon activation, p53 functions as a sequence-specific transcription factor to either activate or repress the expression of target genes. The global landscape of p53 transcriptional regulation is vast and complex, with gene expression profiling studies demonstrating that thousands of genes rapidly alter expression upon p53 activation [1–3]. In addition to direct regulation, p53 also imparts a substantial amount of transcriptional regulation through indirect mechanisms. For example, Nikulenkov *et al*. recently applied a combination of ChIP-Seq (chromatin immunoprecipitation combined DNA sequencing) and RNA-Seq (RNA Sequencing) to demonstrate that, although activation of p53 resulted in the altered transcription levels of over 4500 genes, less than 10% of these genes were directly bound by p53 [2]. Hence, a major component of the p53 transcriptional network is mediated through various indirect effectors or non-protein coding regulators.

MiRNAs (microRNAs) constitutes one of the largest families of trans-acting gene regulators. The discovery that p53 can regulate miRNAs, coupled with the observation that many effects of p53 are indirect, suggests that they could be significant effectors in the p53 transcriptome [4]. Recently, p53 has been shown to transcriptionally activate or repress the expression of several miRNAs, including miR-17-92 cluster [5], miR-22 [6], the miR-34 family [7–11], miR-145 [12], miR-192 family [13, 14], miR-149 [15], miR-200 family [16, 17], miR-605 [18], miR-1204 [19], miR-509 [20], and miR-1915 [21]. Hence, by regulating a miRNA-based network, p53 could modulate an extensive downstream transcriptome. Other families of non-coding RNAs are also emerging as novel entities in the downstream p53 pathway, such as long non-coding RNAs (lncRNAs) [22] and various other pol I/III transcripts including tRNA and rRNAs [23, 24]. The extent to which these and other non-coding RNAs participate in the p53 pathway are currently not well understood.

Herein, we have examined the ability of p53 to regulate miRNA-sized transcripts processed from non-coding small nucleolar RNAs (snoRNAs). Our findings demonstrate a role for p53 in the repression of a family of polycistronic C/D box snoRNAs (SNORDs), of which at least one is processed into an operative miRNA which feeds back to repress TAF9B-mediated stabilization of p53 and promote cell proliferation.

## Results

### Identification of p53 regulated snoRNAs

**Fig 1:**
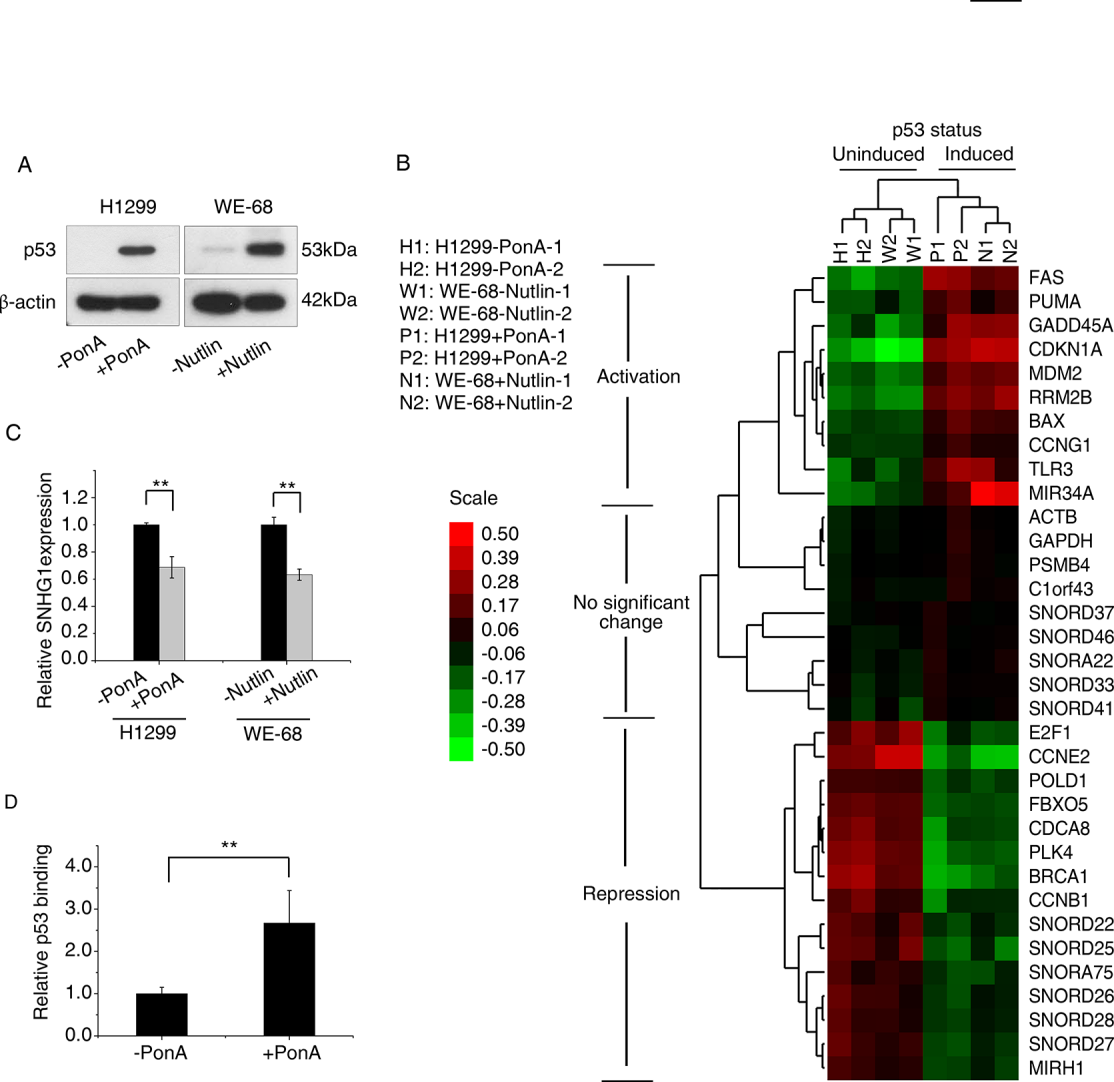
Identification of p53 regulated snoRNAs. (A) Wild type p53-inducible H1299 cells were treated with 2.5μg/ml of Ponasterone A (PonA) for 24 hours, and compared with non-induced cells. WE-68 cells were treated with 10nM Nutlin-3a for 16 hours, compared with non-induced cells. Western blots for p53 (and β-actin as a loading control) are shown. (B) Based on microarray profiling, a dendrogram generated by cluster analysis shows the separation of p53 uninduced cells from induced cells, and separation of representative genes activated, repressed or not significantly changed by p53. (C) SNHG1 expression levels were determined by RT-PCR after p53 induction in H1299 and WE-68 cells. (D) ChIP assay, using the anti-p53 antibody DO-1, was performed to determine relative p53 occupancy in p53-induced H1299 cells (+PonA). RT-PCR results show relative p53 occupation at upstream of the SNHG1 promoter, and p53 null H1299 (-PonA) was used as a negative control. ** p<0.01 versus controls for all experiments.

Given the existing links between p53 and various non-coding RNAs, we hypothesized that p53 may also regulate snoRNAs, as snoRNAs have been linked to carcinogenesis [25, 26] To examine this, we performed snoRNA expression profiling following activation of the p53 signalling network. This was conducted by Affymetrix gene expression profiling in two models of p53 activation to increase the chance of identifying *bona fide* snoRNA targets of p53 that were not restricted to specific cell type, cell origin or mode of p53 activation. These two cell-based models of wild-type p53 activation were (i) an inducible p53 system in the p53 null H1299 cell line (previously characterized in ref. [3]), and (ii) activation of endogenous wild-type p53 through Nutlin-3a treatment of the WE-68 cell line (Fig 1A, S4 Table). Due to space limits, a few representative RNA transcripts are presented in Fig 1B as examples from the microarray to indicate the anticipated responses of these cells to p53 activation, including mRNAs, miRNAs and snoRNAs. According to previous studies, FAS, PUMA (BBC3), CDKN1A, MDM2, RRM2B (p53R2), BAX, CCNG1, TLR3 and MIR34A (miR-34a host gene) are upregulated upon p53 activation [27]; ACTB (β-actin), GAPDH, PSMB4 and C1orf43 are usually stably expressed [28]; whereas E2F1 [27], CCNE2 [29], POLD1 [30], CDCA8 [31], FBXO5 [32], PLK4 [33], BRCA1 [34], CCNB1 [35] and MIRH1 (miR-17-92 host gene) [36] are directly or indirectly repressed upon p53 activation (Fig 1B). Clustering analysis was employed to indicate the relative closeness of the reactive patterns how these genes respond to p53 activation in both cell lines.

**Fig 2:**
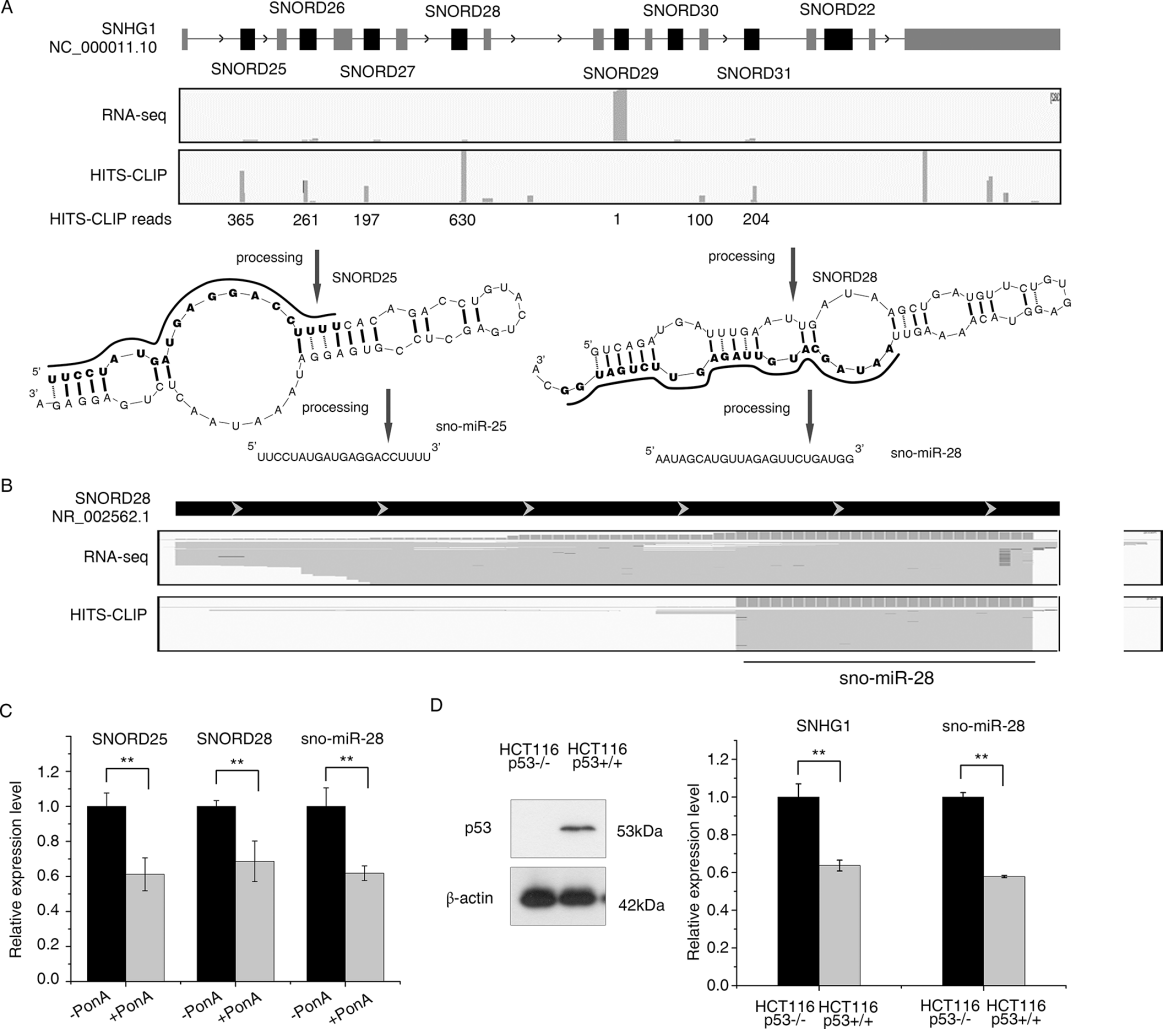
p53 repressed snoRNAs are processed into miRNAs. (A) SNHG1 is processed into snoRNAs including SNORD25 and SNORD28. The top panel shows RNA deep-sequencing and HITS-CLIP (high throughput sequencing of crosslinked and immunoprecipitated RNA) results at the SNHG1 genomic loci. Predicted stem-loop folding of SNORD25 and SNORD28 are shown. The regions marked in bold are processed into sno-miRNAs which can bind to Argonaute proteins, which was confirmed by RNA deep-sequencing and HITS-CLIP results. The solid lines between chains represent hydrogen bonds between adenine (A)-uracil (U) pairs and guanine (G)-cytosine (C) pairs, whereas dashed lines represent G-U pairing. (B) RNA-seq and HITS-CLIP mapping reads across the SNORD28 region is shown indicating precise binding of sno-miR-28 to AGO (C) p53 was induced by PonA treatment in inducible H1299 cells, and the expression levels of SNORD25, SNORD28 and sno-miR-28 were determined using TaqMan assay and RT-PCR. Expression levels of SNORD25, SNORD28 and sno-miR-28 were shown in induced or uninduced cells. (D) Isogenic HCT116 -/-p53 and HCT116 +/+p53 cell lines were used to investigate the relation of SNHG1 and sno-miR-28 expression levels with p53. Left: p53 protein expression in the HCT116 isogenic cell lines was shown by Western blot and β-actin was used as a loading control. Right: SNHG1 and sno-miR-28 expression was determined by RT-PCR as shown. ** p<0.01 versus controls for all experiments.

In a combined analysis of both the H1299 and WE-68 cell lines using Affymetrix microarray, we identified six snoRNAs that were most significantly (fold change > 1.085 or <0.915, p<0.05) regulated by p53 between induced (H1299 treated with PonA and WE-68 treated with Nutlin) and uninduced cells (Fig 1B). Interestingly, all six snoRNAs were clustered to MIRH1, a polycistronic miRNA host gene of the miR-17-92 cluster that is transcriptionally repressed by p53 [36], and this indicates probable similarity in their reactive patterns upon p53 activation (Fig 1B). There were no snoRNAs significantly activated by p53 in either system, suggesting that the role of p53 in the regulation of snoRNAs may be restricted to that of transcriptional repression. Five of these p53-repressed snoRNAs were encoded within the same polycistronic gene, *SNHG1* (SNORD host gene 1), a precursor of a family of C/D box snoRNAs (Table 1, Fig 2A, S1 and S2 Tables). All eight SNHG1-encoded snoRNAs were repressed by p53, with five reaching statistical significance (fold change > 1.085 or <0.915, p<0.05) (Table 1). Consistent with this, upon activation of p53 in both H1299 and WE-68 cell lines, expression levels of SNHG1, the SNORD host gene, was significantly reduced (Fig 1C). We next investigated if SNHG1 was transcriptionally repressed by p53. Analysis of the promoter region of *SNHG1* (~5kB upstream of initiation site of *SNHG1*) using p53 Scan [37] identified a putative p53 responsive element (RE). ChIP (chromatin immunoprecipitation) analysis using the H1299 p53 inducible system indicated modest but reproducible recruitment of p53 to this response element (Fig 1D). Collectively, these data implicate *SNHG1*, and its processed snoRNAs, as p53-repressed targets.

**Table 1:** snoRNAs repressed by p53 in H1299 and WE-68 cells.

Gene Name	Accession Number	p value induced/ uninduced	Chromosome Location	Precursor Transcript
SNORD22	NR_000008	0.021988	11q13	SNHG1
SNORD25	NR_002565	0.019725	11q13	SNHG1
SNORD26	NR_002564	0.036236	11q13	SNHG1
SNORD27	NR_002563	0.018451	11q13	SNHG1
SNORD28	NR_002562	0.02692	11q13	SNHG1
SNORA75	NR_002921	0.010118	2q37.1	NCL

Affymetrix gene expression profiling identified six snoRNAs that were repressed in common in H1299 and WE-68 cell lines as wild-type p53 was induced. Statistical significance of these snoRNAs is characterized by p-values that compare p53 induced (H1299 treated with PonA and WE-68 treated with Nutlin) versus uninduced cells, with their host genes listed.

### p53 repressed snoRNAs are processed into miRNAs

In addition to their well characterized function guiding the enzymatic modification of ribosomal RNA, snoRNAs may also be processed into smaller miRNA-sized molecules which are capable of binding Argonaute (AGO) and exerting miRNA-like effects. These are variously termed sno-miRNAs or snoRNA-derived small RNAs (sdRNAs) [38–40]. Such processing is both widespread and evolutionarily conserved [38], leading us to ask whether the products of SNHG1 may also serve miRNA-like roles.

In order to investigate this, we analysed data from HITS-CLIP (high throughput sequencing of crosslinked and immunoprecipitated RNA) performed in MDA-MB-231 breast cancer cells [41]. Briefly, this technique works by the immunoprecipitation of AGO, a key component of the miRNA processing machinery, with which bound small RNAs are also co-immunoprecipitated. Associated small RNAs are then identified by deep sequencing. We found evidence of miRNA-sized (~17-26nt) molecules derived from all characterized snoRNAs within SNHG1 (Fig 2A), with a specific small RNA mapping to the 3’ end of SNORD28 being the most abundant followed by a small RNA originating from the 5’ end of SNORD25. The SNORD28- and SNORD25- derived small RNAs were sequenced within the library 630 and 365 times respectively, placing them at the level of other moderately-expressed well characterized miRNAs such as miR-155 and miR-34a (Fig 2A). Interestingly, although a small RNA derived from SNORD28 was the most abundant small RNA recruited to AGO, SNORD29 was by far the most abundantly expressed SNHG1-derived snoRNA, demonstrating high processing and/or Ago-binding selectivity (Fig 2A). In addition, the precise 5’ termini of the SNORD-28 derived small RNA further indicates the strong specificity of RNA processing and AGO binding (Fig 2B).

To determine if sno-miR-25 and 28 are really expressed in abundance *in vivo*, we measured the endogenous expression levels of sno-miR-25 and sno-miR-28 in normal and malignant breast tissues, and compared to miR-155 which has a moderate expression level in breast tissues according to miRBase [42]. TaqMan assay showed sno-miR-28 has a higher *in vivo* expression level than miR-155, whereas the expression of sno-miR-25 is extremely low (S2 Fig panel A). For this reason, we focused our research on SNORD28 and sno-miR-28. We confirmed the PCR efficiencies and specificities of the TaqMan assays for these RNAs, demonstrating the sno-miR-28 TaqMan assay is 16 times more specific to sno-miR-28 than to SNORD28 (S1 Fig).

Since SNORD25, SNORD28 and sno-miR-28 are all processed from SNHG1, we hypothesized their expression may be affected through SNHG1 upon p53 activation. Indeed, activation of p53 in H1299 cells resulted in significant downregulation of the expression levels of SNORD25, SNORD28 and sno-miR-28 (Fig 2C). We also demonstrated that this regulatory axis is not restricted to any specific p53 activation models using the HCT116 isogenic cell line system. Indeed, HCT116 (*TP53* ^+/+^) cells express significantly lower levels of SNHG1 and sno-miR-28 than the p53 null HCT116 (*TP53* ^-/-^). Taken together, these results confirm that the SNHG1-sno-miR-28 axis is negatively regulated by p53.

### sno-miR-28 functions as a miRNA

**Fig 3:**
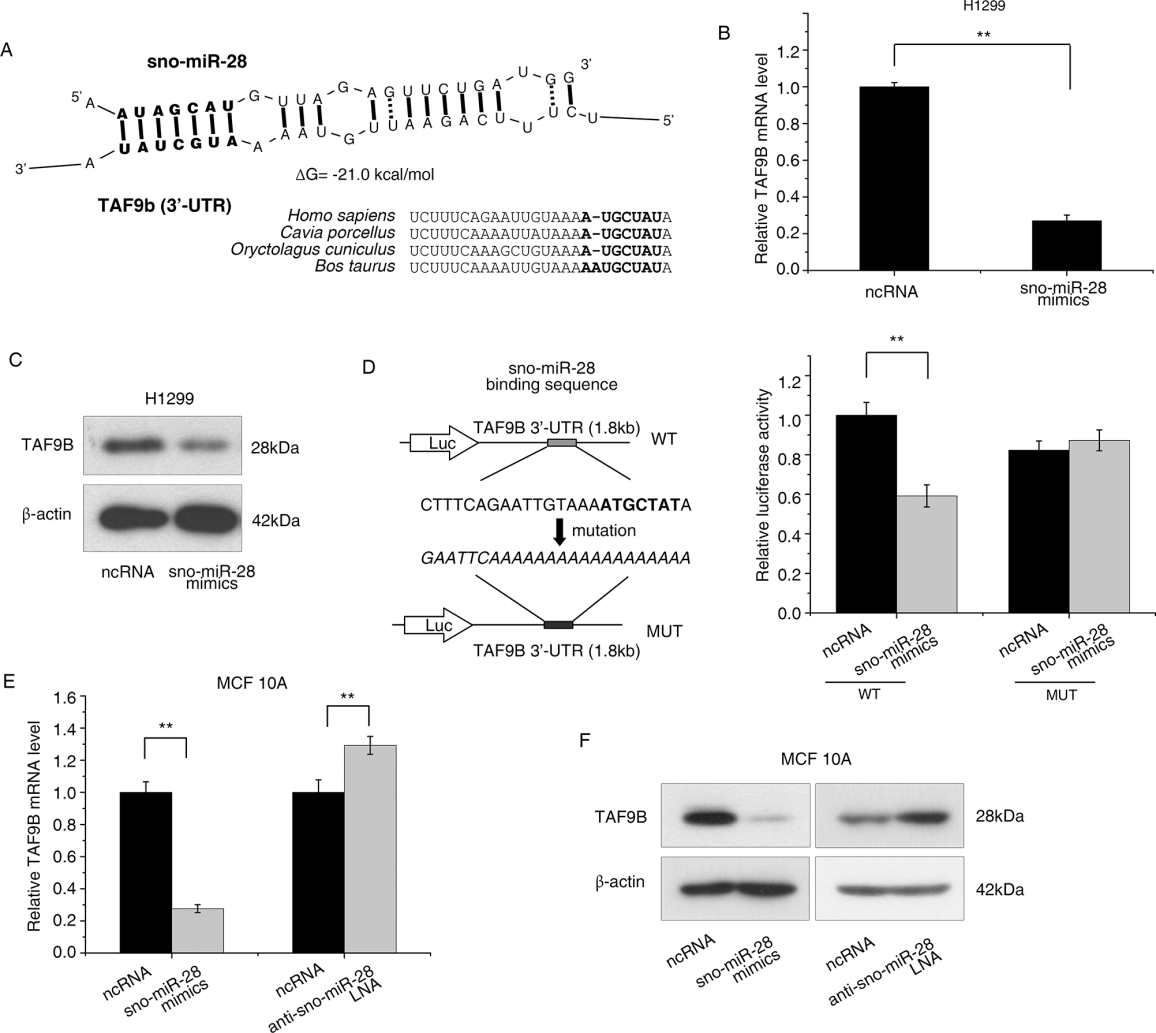
sno-miR-28 functions as a miRNA. (A) Proposed sno-miR-28 binding site within the TAF9B 3’UTR. The seed-recognition site is marked in bold; hypothesized duplexes formed by the interaction of TAF9B and sno-miR-28 are illustrated, and the predicted free energy of the hybrid is indicated. Conservation of the seed region across 4 species is also indicated. (B, C) sno-miR-28 (or negative control RNA, ncRNA) was overexpressed in H1299 cells. TAF9B mRNA and protein levels were determined by RT-PCR and Western blot, respectively. (D) Using a dual-luciferase reporter system, H1299 cells were co-transfected with sno-miR-28 mimics (or negative control RNA), and psiCHECK2 luciferase reporter plasmids with either wild type (WT) or mutated TAF9B 3’-UTR (MUT) cloned at downstream of the Renilla luciferase gene (Luc). Relative luciferase activities are shown. (E, F) sno-miR-28 was either overexpressed (mimics) or inhibited (LNA) in MCF10A cells. TAF9B mRNA levels were determined by RT-PCR (E), and protein expression was determined by Western blot (F). ** p<0.01 versus controls for all experiments, and β-actin was included as a loading control for all Western blots.

Since previous studies have demonstrated miRNA-like functions for sno-miRNAs [38–40, 43–45], we employed a bioinformatics approach to explore potential sno-miR-28 targets. As predicted by TargetScan Custom 5.1, TAF9B (transcription initiation factor TFIID subunit 9B), BHLHE41 (class E basic helix-loop-helix protein 41) and TGFBR2 (transforming growth factor beta receptor II) were identified among the putative targets of sno-miR-28 (Fig 3A, S3 Fig). Several (~10) candidate mRNAs were investigated upon overexpression or inhibition of sno-miR-28 (data not shown), and TAF9B was associated with the greatest level of repression in response to exogenous sno-miR-28. In addition, RNA folding analysis predicted that TAF9B has a moderate-to-high level of hybridization energy binding to sno-miR-28 (ΔG = -21.0 kcal/mol) (Fig 3A). [46]

TAF9B was deduced to be a target of sno-miR-28 not only by bioinformatics analysis, but also by the inverse correlation between sno-miR-28 and TAF9B expression. Following overexpression of sno-miR-28, endogenous TAF9B mRNA and protein expression levels were significantly reduced in H1299 cells (Fig 3B and 3C). To confirm that sno-miR-28 directly interacts with TAF9B’s 3’-UTR (3’-untranslated region), we overexpressed sno-miR-28 along with a psiCHECK2 luciferase reporter containing the TAF9B 3’UTR fused to the 3’ end of the Renilla luciferase gene. We observed that the Renilla luciferase expression was inhibited by sno-miR-28 overexpression, and the repression was abolished by mutation of the proposed sno-miR-28 recognition site (Fig 3D). Taken together, these results demonstrate that sno-miR-28 directly mediates repression of TAF9B through a canonical miRNA binding site.

In order to help verify our observations are not restricted to a specific cell type, the relation between sno-miR-28 and TAF9B was then investigated in MCF10A cells, an immortalized, non-transformed breast epithelial cell line. Transfection of sno-miR-28 mimics downregulates TAF9B mRNA and protein, consistent with sno-miR-28 also functioning like a canonical miRNA in breast epithelial cells (Fig 3E and 3F). Furthermore, a Locked Nucleic Acid (anti-sno-miR-28 LNA) was used to inhibit endogenous sno-miR-28 expression in MCF10A cells and, consistently, TAF9B mRNA and protein expression was increased (Fig 3E and 3F). Taken together, this indicates TAF9B is subject to regulation by the endogenous sno-miR-28.

### sno-miR-28 alters p53 protein stability through TAF9B

**Fig 4:**
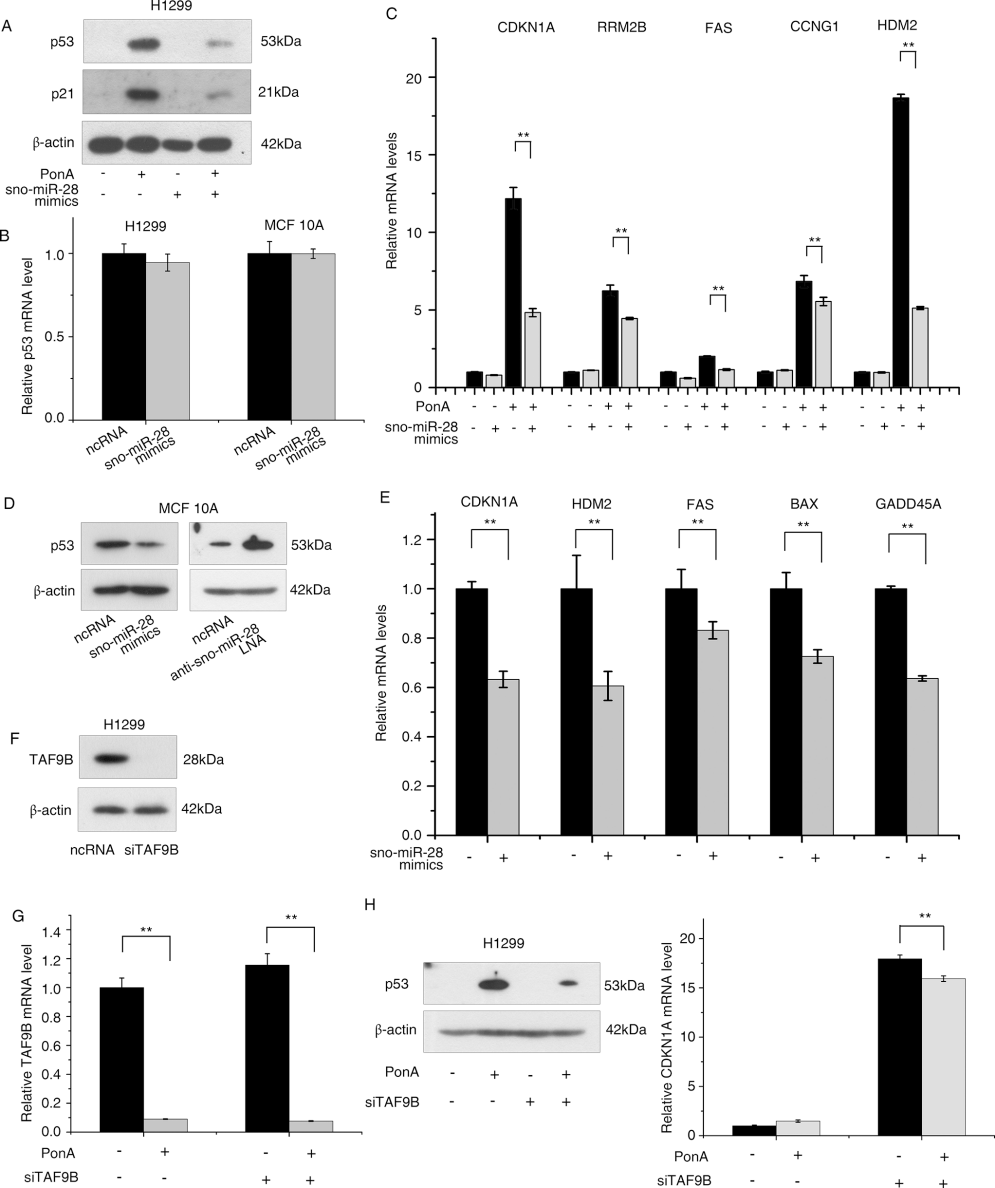
sno-miR-28 alters p53 protein stability through TAF9B and forms a feed-forward loop between p53, sno-miR-28, and TAF9B. (A) Western blots are shown to determine the protein levels of p53 and p21 after overexpression of sno-miR-28 and/or induction of p53 by Ponasterone A (PonA) in inducible H1299 cell line, compared with cells transfected with negative control RNA (ncRNA) and/or uninduced H1299 cells. (B) p53 mRNA levels in MCF10A and PonA-treated H1299 cells after overexpression of sno-miR-28 are shown by RT-PCR, compared with scrambled negative control (ncRNA). (C) The mRNA expression levels of p53 downstream targets are shown after overexpression of sno-miR-28 (or negative control) in H1299 cells upon p53 activation. (D) sno-miR-28 (or negative control) was either expressed (mimic) or inhibited (LNA) in MCF10A cells and the protein expression of p53 was determined by Western blot. (E) The mRNA expression levels of p53 downstream targets are shown after overexpression of sno-miR-28 in MCF10A cell line, compared with ncRNA. (F,G) TAF9B was knocked using an siRNA in H1299 cells compared with a negative control. Successful knockdown is shown at both the protein (F) and mRNA (G) levels. (H) CDKN1A expression was determined by RT-PCR after TAF9B was knockdown. ** p<0.01 versus controls for all experiments. β-actin is included as a loading control for all Western blots. “-”mark in sno-miR-28 mimic and anti-sno-miR-28 LNA experiments represent negative control transfections using a scrambled ncRNA.

TAF9B functions as a subunit of TFIID (transcription initiation factor II D) and TFTC (TATA-binding Protein-free TAF-containing) complexes. It also acts as a p53 co-activator, stabilizing p53 possibly by competing for Mdm2 binding [47, 48]. We therefore reasoned that via its direct regulation of TAF9B, sno-miR-28 may indirectly regulate p53. To investigate this, we examined p53 protein levels after sno-miR-28 overexpression in H1299 cells and found that sno-miR-28 downregulated p53 protein but not RNA (Fig 4A and 4B), suggesting the sno-miR-28 and TAF9B regulation of p53 may function at the protein level. In addition, sno-miR-28 overexpression also significantly repressed multiple p53 regulated genes including CDKN1A (p21), RRM2B, CCNG1, FAS and HDM2 in induced H1299 cells (Fig 4A and 4C).

To confirm that the sno-miR-28-TAF9B-p53 regulatory axis is not restricted to a specific cell type or model of p53 activation, we also investigated this pathway in MCF10A cells. As seen in H1299 cells, sno-miR-28 overexpression reduced p53 protein but not mRNA in MCF10A cells, while inhibition of sno-miR-28 restored p53 protein levels (Fig 4B and 4D). In addition, overexpression of sno-miR-28 repressed the mRNA levels of various p53 downstream regulators including CDKN1A, HDM2, FAS, BAX and GADD45A (Fig 4E) highlighting the prevalent influence of sno-miR-28 in p53 signalling.

Consistent with sno-miR-28 stabilizing p53 protein via its regulation of TAF9B, we found that siRNA-mediated knockdown of TAF9B phenocopied the effect of sno-miR-28, reducing the levels of p53 protein and CDK1A mRNA (Fig 4F–4H).

### sno-miR-28 is upregulated in breast tumours and promotes breast cell proliferation

**Fig 5:**
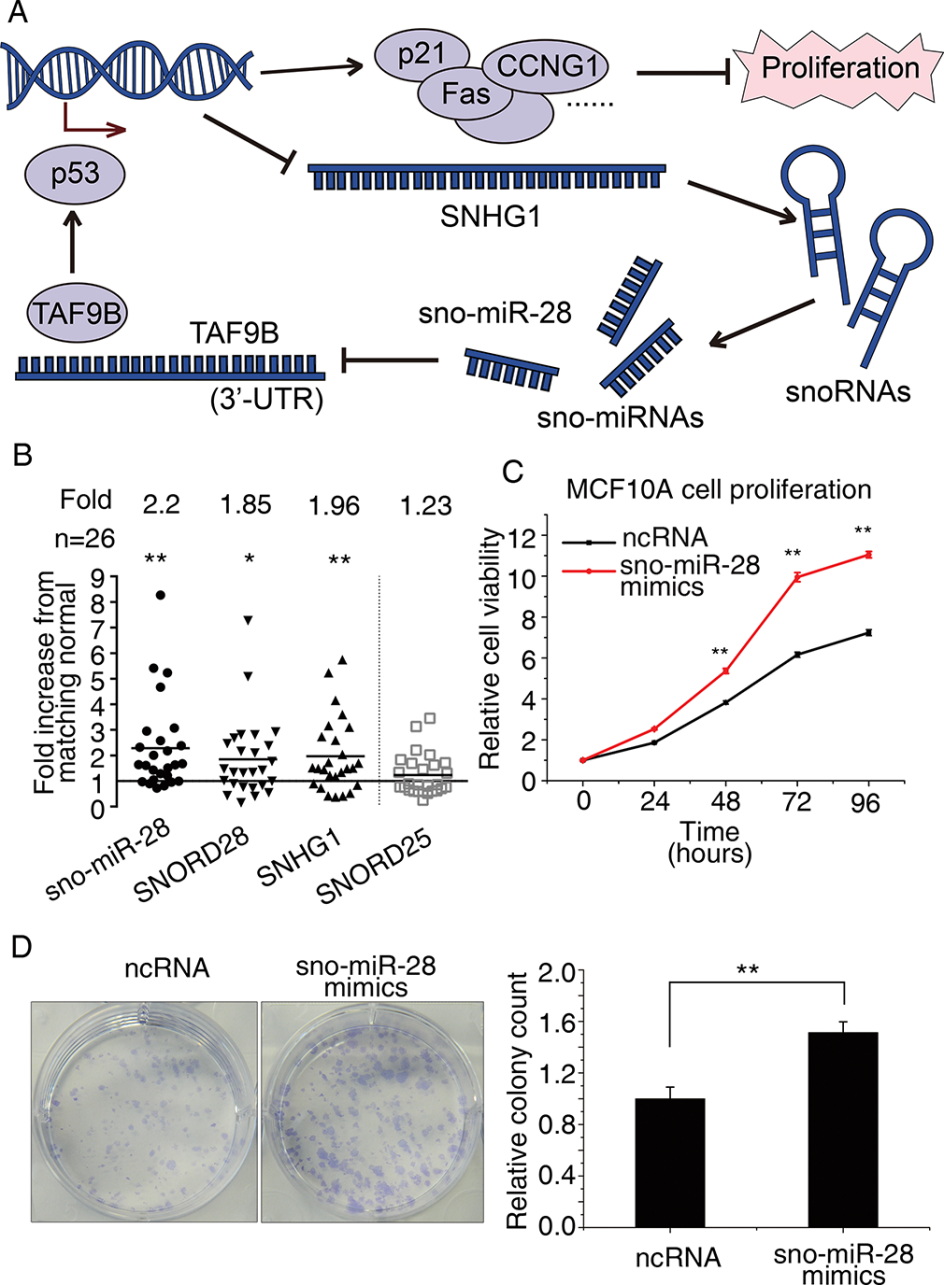
sno-miR-28 is over-expressed in breast tumours. (A) The feed-forward loop between p53, sno-miR-28, and TAF9B is shown as proposed. (B) sno-miR-28, SNORD28, and SNORD25 expression levels were determined using TaqMan assay in breast tumours compared with paired normal adjacent tissues, while SNHG1 expression levels were determined by RT-PCR. In this part of figure, RNA expression levels are shown as the ratio relative to normal tissue expression; e.g., 1 represents equal expression to normal tissues. (C) MCF10A cell proliferation is shown after overexpression of sno-miR-28 compared with negative control RNA (ncRNA). (D) Colony formation assay in MCF10A cells after expression of sno-miR-28 or a negative control RNA (ncRNA). Representative images are included on the left, and relative quantitation of the graph is on the right. ** p<0.01 and * p≤0.1 versus controls for all experiments.

Our findings indicate that sno-miR-28 participates in a feed forward loop with p53, whereby p53 represses sno-miR-28 via SNHG1, whilst sno-miR-28 directly targets the TAF9B 3’UTR to negatively regulate p53 stability (Fig 5A). The reciprocal negative association with p53 implies sno-miR-28 might have an oncogenic role. Consistent with this, SNHG1 is upregulated in gastric cancer [49]. To further investigate, in a cohort of 26 pairs of matched malignant and non-malignant breast tissues samples, we found that SNHG1, SNORD28 and sno-miR-28 were all significantly upregulated in breast tumours (Fig 5B). Two well-characterized oncogenic miRNAs, miR-21 and miR-155, that were used as positive controls were similarly upregulated (S2 Fig panel B) [50, 51]. In further agreement with an oncogenic role for sno-miR-28, we also found that sno-miR-28 expression promoted the proliferation (Fig 5C) and colony forming capacity (Fig 5D) of MCF10A cells. Taken as a whole, these results demonstrate that sno-miR-28, which targets TAF9B, antagonizes p53 protein levels and is capable of playing an oncogenic role to accelerate breast cell proliferation and colony formation.

## Discussion

### snoRNAs are processed into miRNAs

Through the use of both microarrays and the analysis of endogenous small RNAs bound to AGO, we discovered that a number of polycistronic snoRNAs are processed into miRNA-sized molecules from a common host gene (SNHG1). Interestingly, we noticed from whole-cell RNA-seq that SNORD29 is far more abundant than SNORD28, but in contrast, the sno-miRNA derived from the SNORD28 region is the most abundantly recruited to AGO (Fig 2A). The 5’ end of sno-miR-28 is also very precisely processed, indicating that the processing and recruitment of snoRNA-derived products to AGO can be highly selective [52]. In addition, up-regulation of SNORD28 but not SNORD25 in breast cancer tissues (Fig 5B) also indicates existence of extensive posttranscriptional regulation. These evidences strongly imply the presence of a *bonda fide* RNA product, rather than random RNA degradation debris. This provides another example of concomitant post-transcriptional regulation of miRNA levels by regulating their precursors. For instance, LIN28 regulates miR-9 by inducing degradation of its precursor [53], and blocks processing of let-7 by co-transcriptional binding [54]. In accordance with our data, other researchers have also found smaller processed products of snoRNAs to have miRNA-like functions. For example, snoRNA ACA45 is processed to small 20- to 25-nt-long RNAs, one of which regulates the 3’-UTR of CDC2L6 mRNA by stably associating with AGO proteins [44], and 11 box C/D sno-miRNAs were found to have efficient gene silencing function [39]. In addition, computational analyses has identified 84 intronic miRNAs that are encoded within either box C/D snoRNAs, or in precursors showing similarity to box C/D snoRNAs [40]. Whilst functions for most of these small RNAs have not been determined, the expanded use of deep sequencing technologies have led to the extensive profiling of small RNA fragments derived from a number of sources, including snoRNAs, tRNAs and rRNAs, and the characterization of their dependence on Drosha or Dicer for their production [55].

### sno-miR-28 is a novel member of the p53 regulatory network

While miRNAs have been known to play vital roles in the p53 pathway, our research has revealed a novel miRNA regulatory pathway based on a sno-miRNA in p53 regulation. We discovered that p53 transcriptionally regulates the host gene of sno-miR-28 which targets TAF9B. TAF9B is a subunit of TFIID and TAFIIC, functioning as a stabilizer and co-activator of the p53 protein, and has been reported to be essential for cell viability [47, 48]. In addition, TAF9B has been previously discovered to play a role in transcriptional repression and silencing [56]. The regulation of TAF9B by sno-miR-28, and the reciprocal repression of the sno-miR host gene by p53, suggests a role for sno-miR-28: p53 feedback in cancer, which supports recent discoveries that a large number of miRNAs interact with the p53 network as an alternative mechanism of the tumour-suppressor activity of p53 [5–17, 19–21, 57]. The complex regulatory loop involving p53, SNHG1 and TAF9B is also reminiscent of the feed-back and feed-forward motifs with which miRNAs are frequently associated [20, 58].

Furthermore, we noticed that sno-miR-28 actually represses CDKN1A mRNA more effectively than merely knocking down TAF9B using a siRNA (Fig 4A, 4C, 4E and 4H), and this might be explained by the fact that miRNAs usually have a large number of targets, many of which have synergistic functions in the same pathway. For instance, miR-34a targets E2F3 [59], CCNE2 and CDK4 [9], CCND1 and CDK6 [60] in cell cycle regulation, and no single target can fully cover its entire function. We anticipate there will be a large number of additional sno-miR-28 targets awaiting discovery.

### sno-miR-28 is overexpressed in breast tumour samples and plays an oncogenic role in breast cells

The regulatory role of sno-miR-28 is further confirmed by our expression profiling studies which relate SNHG1, SNORD28 and sno-miR-28 to breast tumours. Interestingly, SNHG1 was also reported as one of the 5 most significantly upregulated long non-coding RNAs (lncRNAs) in gastric cancer among the 9294 lncRNAs detected [61]. The upregulation of sno-miRNA-28 and SNHG1 in tumours and its relationship to p53 parallels our understanding of other miRNA regulatory loops that closely interact with the p53 pathway and play significant roles in p53-mediated apoptosis and cell cycle arrest. For example, the miR-34 family mediates tumour suppression through a positive feedback loop involving p53 and MDM4 [62]; 15 miRNAs, including the miR-106b/93/25 cluster, miR-17-92 cluster and the miR-106a-92 cluster, are repressed by p53 and involved with E2F in a feed-forward loop promoting proliferation [63]; whereas miR-192, 194 and 215 are involved in the p53-MDM2 auto-regulatory loop [64]. Whilst our research has revealed yet another feed-forward loop involving p53 and a miRNA, it is surprising that this regulatory loop is based on a snoRNA-derived miRNA, thereby building upon the novel regulatory roles of snoRNAs in cancer.

Collectively, this research builds on a small but growing knowledge base of small RNAs from “unfamiliar” sources playing important biological roles. We believe more novel targets of sno-miR-28 will be identified, and functions for more miRNAs derived from novel sources will be explored. From this point of view, developments of knowledge in cancer regulatory networks and their interaction with small non-coding RNAs may eventually offer insights into new approaches to cancer treatment.

## Materials and Methods

### HITS-CLIP (High throughput sequencing of crosslinked and immunoprecipitated RNA):

MDA-MB-231 cells were suspended in cold PBS by scraping cross-linked at 254 nM using a Stratalinker. Cell pellets were lysed (0.1% SDS, 0.5% deoxycholate, 0.5% NP-40 with protease inhibitors, Roche, Indianapolis, IN USA) for 10 mins on ice followed by RQ1 DNAse (Promega, Madison, WI USA) at 37°C for 15 mins with shaking. RNAse A/T1 (Ambion, Grand Island, NY USA) was then added for 8 minutes, prior to the addition of EDTA (30 mM). Pellets were then spun (30,000 rpm) and the lysate subjected to immunoprecipitation for 2 h with a pan-anti-AGO antibody (2A8) conjugated to protein-A dynabeads (Invitrogen, Grand Island, NY USA) using bridging rabbit anti-mouse IgG (Jackson ImmunoResearch Laboratories, West Grove, PA USA). Pellets were then successively washed (0.1% SDS, 0.5% deoxycholate, 0.5% NP40 in 1 × PBS; 0.1% SDS, 0.5% deoxycholate, 0.5% NP40 in 5 × PBS; 50 mM Tris pH 7.5, 10 mM MgCl_2_, 0.5% NP40) and on-bead phosphatase treatment performed for 30 mins with antarctic phosphatase (New England Biolabs, NEB, Ipswich, MA USA) in the presence of superasin RNAse inhibitor (Ambion). The 3’ RNA linker (CAGACGACGAGCGGG) was labeled with P^32^ using T4-PNK (NEB) and ligated on-bead for 1 h at 16°C with T4 RNA ligase (Fermentas, Thermo Fisher Scientific, Pittsburgh, PA USA). Beads were then washed as previous and treated with PNK to ligate the 5’ RNA linker (AGGGAGGACGAUGCGGxxxG, with “X” representing different nucleotides for barcoding). Beads were resuspended in 4 × LDS Novex loading buffer with 4% B-mercaptoethanol, incubated at 70°C for 10 mins and the supernatant loaded on Novex NuPAGE 4–12% Bis-Tris acrylamide gels (Bio-Rad, Hercules, CA USA). After running, the Ago-RNA complexes were then transferred to nitrocellulose and exposed to film at -80°C for 3 days. Complexes running at ~110 kDa were then excized with a scalpel and resuspended (100 mM Tris pH 7.5, 50 mM NaCl, 10 mM EDTA, 4 mg/ml proteinase K) for 20 min at 37°C. The sample was incubated for an additional 20 minutes in the presence of 3.5 M urea and RNA isolated by a phenol-chloroform extraction. Samples were then run on a 10% denaturing (1:19) polyacrylamide gel and exposed to film with an intensifying screen at -80°C for 5 days. A thin band corresponding to the Ago-miRNA (~110 kD) was excized, crushed and eluted at 37°C for 1 h (1M NaOAc, pH 5.2, 1 mM EDTA). RNA was precipitated overnight with ethanol, centrifuged and dried, and then resuspended in 8 μl H_2_O. Next, reverse transcription primer was added and reverse transcription performed using SuperScriptIII (Invitrogen). After that, PCR was performed with the above primer and a reverse primer for 25 cycles, and PCR product was run on a 10% native (1:29) polyacrylamide gel, stained with Sybr Gold (Qiagen, Valencia, CA USA) and bands excized over a UV light box. Following this, the DNA was precipitated using isopropanol and a final 10-cycle PCR performed with the HITS-CLIP primers listed in S3 Table. Reactions were subsequently run on 2% metaphor agarose/TBE gels and bands (~115 bp) excized corresponding to the linker sequence + miRNA CLIP tag. Samples were finally purified using quick-spin columns (Qiagen) and subjected to Illumina sequencing (Illuminia, San Diego, CA USA). Using an in house Perl script, reads were filtered for average quality and for homopolymeric tracts exceeding 12 nt, trimmed of linker sequence fragments and separated by barcode. The bowtie program [65] was used to align resulting 17 to 30 nt reads to the human genome. The dataset has been previously published [41] and has been deposited at the NCBI (National Center for Biotechnology Information) SRA (Sequence Read Archive) SRP045204, BioProject PRJNA257235.

### Cell lines and reagents

HCT116 (human colon cancer) and its *TP53* ^–/–^derivative were supplied by B. Vogelstein [66]; MDA-MB-231 (human breast cancer) and MCF10A (human breast epithelial) cells were purchased from ATCC (American Type Culture Collection, Manassas, VA USA); wild-type p53-inducible H1299 (mentioned as H1299 below) human non-small cell lung cancer cell line was generated as previously described [67] and was characterized in Fig 1A using Western blot; WE-68 human Ewing's sarcoma cell line was a kind gift from Prof F. van Valen (Department of Orthopaedic Surgery, Westfälische-Wilhelms-University, Germany) as previously described [68]. HCT116, MDA-MB-231 and H1299 cell lines were cultured in DMEM (Sigma-Aldrich, St. Louis, USA) and WE-68 cells were cultured on type-1 collagen-coated plates (Iwaki, Thermo Fisher, Newport, UK) in RPMI-1640 (Sigma-Aldrich). The media above were supplemented with 10% (v/v) fetal calf serum and 10mmol/L HEPES. MCF10A cells were cultured in DMEM/F12 medium (Sigma-Aldrich) supplemented with 5% horse serum, 20ng/mL EGF, 0.5μL/ml Hydrocortisone, 100ng/ml Cholera toxin, 10μg/mL Insulin. All media were supplemented with 2mmol/L L-Glutamine, 100 IU/mL penicillin and 100μg/ml streptomycin, and all cell lines were cultured at 37°C in a humidified atmosphere at 5% CO_2_. All cell lines have been under regular tests every 1–2 months using MycoAlert mycoplasma detection kit (Lonza, Melbourne, VIC Australia), which ensured that all cell lines being used for experiments were safe from mycoplasma contamination.

Nutlin-3a (4-[[(4S,5R)-4,5-bis(4-chlorophenyl)-4,5-dihydro-2-[4-methoxy-2-(1-methylethoxy)phenyl]-1H-imidazol-1-yl]carbonyl]-2-piperazinone) was purchased from Cayman Biochemicals (Ann Arbor, MI USA), and Ponasterone A (PonA) was purchased from Invitrogen.

### Breast tumour and paired normal tissues

Breast tumour and paired normal adjacent tissue samples were obtained from 26 breast cancer patients who have received surgery at the Royal Adelaide Hospital (Adelaide, Australia) between 2003 and 2011. Samples were stored in RNAlater solution (Ambion) for RNA stabilization before RNA extraction using miRNeasy kit (Qiagen) following the manufacturer’s protocol. RNA concentration was determined using a ND-1000 NanoDrop spectrometer (Thermo Scientific, Wilmington, DE USA). This research has been approved by the Institutional Review Boards of the University of Adelaide and SA Pathology. All samples were gathered according to the institutional review board-approved protocol and the written informed consent from each patient. Relevant clinical data was retrieved from patients’ records including human epidermal growth factor 2 (HER2), estrogen receptor (ER), progesterone receptor (PR) and proliferation index (MIB-1) status.

### miRNA overexpression and inhibition

For miRNA overexpression studies, approximately 3 × 10^5^ cells/well were seeded in 6-well plates (for RT-PCR (real-time quantitative PCR) or Western blot) and 1 x 10^5^ cells/well were seeded in 24-well plates (for luciferase assay, proliferation assay and colony formation). Transfections were done 24 hours post-seeding with 50 nM Genepharma miRNA mimics (Genepharma, Shanghai, China) or negative control RNA (ncRNA) (Genepharma) using Lipofectamine RNAiMAX reagent (Invitrogen) following the manufacturer’s instructions. The cells were harvested 72 hours post-transfection for RT-PCR, Western blot, or luciferase assay.

For miRNA inhibition studies, approximately 3 × 10^5^ cells/well were seeded in six-well plates. The first transfection was done 24 hours post-seeding with 50 nM miRVana miRNA inhibitor (Ambion) or 50 nM miRVana miRNA inhibitor negative control RNA (ncRNA) (Ambion) using Lipofectamine RNAiMAX reagent (Invitrogen) following the manufacturer’s instructions. Cells were harvested 72 hours post-transfection for further experiments.

### RNA interference

RNA interference of TAF9B was performed using siTAF9B (AGUAUGAACCAAGGGUUAUAA) (GenePharma). The transfection procedure using Lipofectamine RNAiMAX reagent (Invitrogen) is the same as described above for miRNA overexpression and inhibition.

### Luciferase Assay

For the luciferase assay, we cloned the TAF9B 3’-UTR (WT) downstream of the Renilla luciferase gene in the psiCHECK2 dual-luciferase vector (Promega) (for primers, see S3 Table). The Firefly luciferase gene (which is expressed from the same vector from an HSV-TK promoter) was used as an internal reference. For the mutated TAF9B 3’-UTR (MUT) construct, a 24 bp mutation was introduced at the proposed sno-miR-28 binding site (CTTTCAGAATTGTAAAATGCTATA to GAATTCAAAAAAAAAAAAAAAAAA) (Fig 3D, left panel) (for primers, see S3 Table). Approximately 1 × 10^5^ H1299 cells/well in 24-well plates. After 24 hours, in each well we co-transfected 0.4 ng/μL of either WT or MUT luciferase constructs with 8.33 nM of either sno-miR-28 mimics (Genepharma) or negative control RNA (ncRNA) (Genepharma), using Lipofectamine 2000 (Invitrogen) following the manufacturer’s instructions. Cells were harvested 72 hours post-transfection, and Renilla and Firefly luminescence were measured by a GloMax 20/20 Luminometer (Promega) following the manufacturer’s instructions. A ratio of Renilla/Firefly luminescence intensity was used to indicate the relative luciferase expression activity.

### Western blot and ChIP analysis

Cells were rinsed with PBS and lysed in lysis buffer (50 mmol/L Tris-HCl, pH 7.5, 250 mmol/L NaCl, 1% Triton X-100 and 1x protease inhibitors (Roche, South San Francisco, CA)) on ice for 30 min. Insoluble components of cell lysates were removed by centrifugation for 10 min at 4°C, 12,000 *g*, and protein concentration was measured using a bicinchoninic acid protein assay kit (Pierce, Thermo Scientific, Radnor, PA USA). Protein extracts were resolved using SDS PAGE electrophoresis on 10% polyacrylamide gels and electrotransferred to Hybond- C Extra nitrocellulose membranes (GE Healthcare Life Sciences, Pittsburgh, PA USA).

For quantification, p53 protein was probed by mouse monoclonal p53 antibody (DO-1) (Santa Cruz, 1:1000) (Dallas, TX USA); TAF9B was probed by rabbit monoclonal TAF9B antibody (Abcam, at 1:1000) (Cambridge, MA USA); p21 was probed by mouse monoclonal p21^WAF1^ Ab-3 antibody (Thermo Scientific, 1:250); Equal loading was confirmed by blotting of β-actin antibody (Sigma-Aldrich, 1:2000). Chemiluminescent detection of protein was done using appropriate secondary antibodies conjugated with horseradish peroxidise (GE Healthcare) and the enhanced chemiluminescence kit (GE Healthcare) according to the manufacturer's instructions.

ChIP analysis was performed as previously described [57] using mouse p53 antibody (DO-1) (Santa Cruz). The uninduced H1299 cells were used as a p53-null control. Levels of specific promoter DNAs were determined by real-time PCR using specific primers. A negative genomic region which does not contain any p53 responsive element was used as a negative control, and a pair of primers targeting the p53 recognition site of *CDKN1A* was used as a positive control (S3 Table). Data presented is the mean of three independent biological replicates.

### RNA extraction and Real-time PCR

RNA (>250nt) extraction from cells was performed using the RNAeasy mini kit (Qiagen) using on-column RNase-free DNase digestion according to the manufacturer’s instructions. After extraction, RNA concentration was measured using a ND-1000 NanoDrop spectrometer (Thermo Scientific). Thereafter, 1μg of total RNA was reverse-transcribed into cDNA using Moloney Murine Leukaemia Virus (M-MLV) Reverse Transcriptase (Promega) with random 6’mer primers or oligo-dT primer (Promega) under the following the manufacturer’s instructions. Real-time PCR reaction was performed on a CFX Connect Real-Time PCR Detection System (Bio-Rad) using iTaq Universal SYBR Green Supermix (Bio-Rad). The RT-PCR program was: 95°C for 3min, then start cycles consisting 95°C for 10s and 57/60/61°C for 60s (depending on the primers used) for 40 cycles. After the reactions were complete, the C_T_ values were determined using automated threshold settings. In this study, RNA (>250bp) expression was normalized to PSMB4 (S3 Table).

Small RNAs (including miRNAs and sno-miRNAs) were extracted using the miRNeasy mini kit (Qiagen) following the manufacturer’s instructions. After extraction, total RNA concentration was determined using a ND-1000 NanoDrop spectrometer (Thermo Scientific). For quantification of small RNAs, TaqMan gene expression assays were obtained from Applied Biosystems (Grand Island, NY USA), including TaqMan assays for miR-21, miR-155, miR-16, miR-24 and U6 snRNA, and Custom TaqMan assays for SNORD25, SNORD28, sno-miR-25, sno-miR-28. Reverse transcription was performed using a TaqMan MicroRNA Reverse Transcription Kit (Applied Biosystems) and specific Reverse Transcription stem-loop primers provided in the TaqMan RNA assays following the manufacturer’s instructions. Real-time PCR reaction was performed on a Rotor Gene 6000 Real-Time PCR Machine (Qiagen) using specific TaqMan RNA assays following the manufacturer’s instructions. Small RNA expression in cell samples was normalized to U6 snRNA, whereas small RNA expression in tissue samples was normalized to averaged relative expression level of a group of pooled normalizers: miR-24, U44, U48 and U6.

To determine the endogenous expression levels of sno-miRNAs in breast tissues, we performed absolute quantitation of sno-miR-28 and sno-miR-28 in breast tissues, and miR-155 was used as a positive control. Synthesized sno-miR-28 and miR-155 miRNA oligos were prepared at a gradient of known concentrations at 4 nM, 0.4 nM, 0.04 nM and 0.004 nM, while synthesized sno-miR-25 was prepared at 4 pM, 0.4 pM, 0.04 pM and 0.004 pM to match its lower expression in tissues. RT-PCR was performed subsequently and C_T_ values were plotted to log_10_ of the miRNA concentrations respectively to create a standard curve (S1 Fig panels A, B, C), which was used to determine the endogenous expression levels of sno-miR-28, sno-miR-25 and miR-155 in 26 breast tumour tissues and paired-matching non-malignant breast tissues (S2 Fig panel A).

Since sno-miR-28 is processed from a region of SNORD28 near its 3’ end (Fig 2A), there is possibility of mispriming in stem-loop PCR. To make sure that our sno-miRNA expression data is not a result of mispriming, we conducted sno-miR-28 TaqMan RT-PCR using 2nM of synthesized SNORD28 mimics or sno-miR-28 mimics as templates.

### Cell proliferation assay and colony formation

MCF10A breast cells were plated in 24-well plates at about 1 x 10^5^ cells/well and transfected according to the procedure described above as miRNA overexpression. At 72 hours post-transfection, the cells were harvested for proliferation or colony formation assays.

For proliferation assay, the cells were re-plated in 96-well plates in 5 replicate wells for each experimental group (i.e., ncRNA, sno-miR-28 mimics) at 2000 cells/well and were incubated in normal culture conditions for 6 hours to attach. After that, these cells were processed at 0, 24, 48, 72, 96 hour time points using the CellTiter-Glo Luminescent Cell Viability Assay Kit (Promega) according to the manufacturer’s instructions. Fluorescence was measured using a LUMIstar Galaxy luminometer (BMG Labtech). The fluorescence readings in each experimental group were normalized against the 0 hour point.

For colony formation, cells were re-plated in 6-well plates at 500/1000/2000 cells/well and were incubated in normal culturing conditions for 7 days. Then the cells were fixed in methanol for 15 minutes, and stained for 1 hour in 1:20 Giemsa Stain, Modified Solution (Sigma-Aldrich). Afterwards, colonies were photographed by a digital camera and counted.

### Microarray profiling

An Affymetrix Human Gene 1.0 ST array containing 234 annotated snoRNAs (Affymetrix, Santa Clara, CA USA) was used to identify snoRNAs that were differentially expressed following the manufacturer’s instructions as previously described [57, 69]. “Fold change” refers to the ratio of expression in induced (H1299 treated with PonA and WE-68 treated with Nutlin) versus uninduced cells. Statistical criteria for microarray: fold change > 1.085 or <0.915, p<0.05.

### Statistical analysis

Results are given as mean of at least three independent experiments ± standard deviation. Student’s t-test was performed using replicate values to indicate significance. Values of p<0.05 were considered statistically significant (as labelled with ** in figures), while values of p<0.1 were indicated by *.

## Supporting Information

S1 Fig Characterization of snoRNA and sno-miRNA RT-PCR. (A, B, C) Standard curves were shown for absolute quantitation of endogenous sno-miR-28, sno-miR-25 using custom TaqMan assays with miR-155 included as a positive control. Synthesized sno-miR-28 and miR-155 were prepared at a series of known concentrations at 4nM, 0.4nM, 0.04nM, and 0.004 nM, while synthesized sno-miR-25 was prepared at 4pM, 0.4pM, 0.04pM, and 0.004 pM to match its low endogenous expression detected in tissues. RT-PCR was performed subsequently and CT values were plotted to log10 of the miRNA concentrations. The point representing 0.004pM of sno-miR-25 was not plotted because no CT value was detectable at this concentration. (D) sno-miR-28 TaqMan RT-PCR was performed using synthesized SNORD28 or sno-miR-28 as templates. RT-PCR readings are shown to evaluate the specificity of the sno-miR-28 TaqMan assay.
(TIF)

S2 Fig Evaluation of endogenous expression of miRNAs. (A) The standard curves in S1 Fig were used to define a standard curve for the absolute expression of miR-155, sno-miR-28 and sno-miR-25 in 26 breast tumour tissues and paired normal adjacent breast tissues using TaqMan assay and RT-PCR. The average expression levels of sno-miR-28, miR-155, and sno-miR-25 are shown in millions of molecules per nanogram (ng) of total RNA. (B) miR-21 and miR-155 expression was determined as positive controls using Taqman assay and RT-PCR in breast tumours compared with paired normal tissue.
(TIF)

S3 Fig Hybridization of sno-miR-28 binding to BHLHE41 (A) and TGFBR2 (B). Bioinformatics analysis showed the binding of sno-miR-28 to BHLHE41 and TGFBR2 3’-UTR in a similar manner as other miRNAs binding to their targets. The schematic graphs are made to show the proposed binding sites for sno-miR-28. The seed-recognizing sites are marked in red; hypothesized duplexes formed by the interaction of the binding sites of the 3’-UTR of TAF9B and sno-miR-28 are illustrated, and the predicted free energy of the hybrids were indicated. The solid lines between two chains represent hydrogen bonds between adenine (A)-uracil (U) pairs and guanine (G)-cytosine (C) pairs, whereas dashed lines represent G-U pairings.
(TIF)

S1 Table snoRNAs repressed by p53 in H1299 cells. As wild-type p53 was induced in H1299 cells, Affymetrix gene expression profiling identified a list of snoRNAs that were repressed. These snoRNAs are listed with their host genes.
(PDF)

S2 Table snoRNAs repressed by p53 in WE-68 cells. When wild-type p53 was induced in WE-68 cells, Affymetrix gene expression profiling identified a list of snoRNAs that were repressed. These snoRNAs are shown with their host genes.
(PDF)

S3 Table Primer list. Primers used in this study are listed by targets and usage.
(DOC)

S4 Table Significant genes in the microarray. All genes significantly regulated in both H1299 and WE-68 cell lines upon p53 activation are listed with fold change and p values. “Fold change” refers to the ratio of expression in induced (H1299 treated with PonA and WE-68 treated with Nutlin) versus uninduced cells. Statistical criteria for microarray: fold change > 1.085 or <0.915, p<0.05.
(XLSX)
