# Supplementary figures and images for: p53 Represses the Oncogenic Sno-MiR-28 Derived from a SnoRNA
Source: PLoS One. 2015 Jun 10;10(6):e0129190. doi: 10.1371/journal.pone.0129190 (PMC4465335; doi:10.1371/journal.pone.0129190)

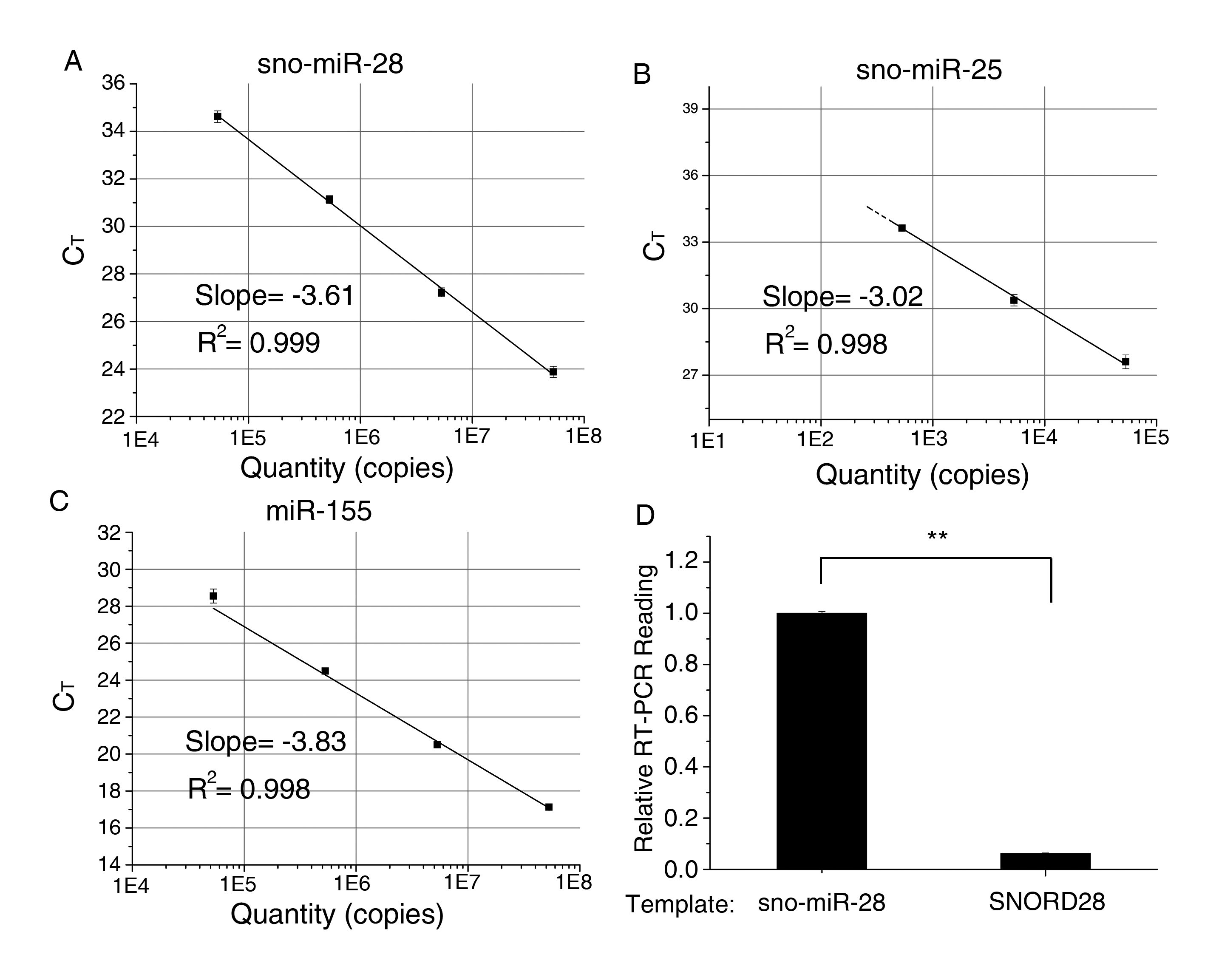

Supplement: S1 Fig — (A, B, C) Standard curves were shown for absolute quantitation of endogenous sno-miR-28, sno-miR-25 using custom TaqMan assays with miR-155 included as a positive control. Synthesized sno-miR-28 and miR-155 were prepared at a series of known concentrations at 4nM, 0.4nM, 0.04nM, and 0.004 nM, while synthesized sno-miR-25 was prepared at 4pM, 0.4pM, 0.04pM, and 0.004 pM to match its low endogenous expression detected in tissues. RT-PCR was performed subsequently and CT values were plotted to log10 of the miRNA concentrations. The point representing 0.004pM of sno-miR-25 was not plotted because no CT value was detectable at this concentration. (D) sno-miR-28 TaqMan RT-PCR was performed using synthesized SNORD28 or sno-miR-28 as templates. RT-PCR readings are shown to evaluate the specificity of the sno-miR-28 TaqMan assay. (TIF) [file pone.0129190.s001.tif]

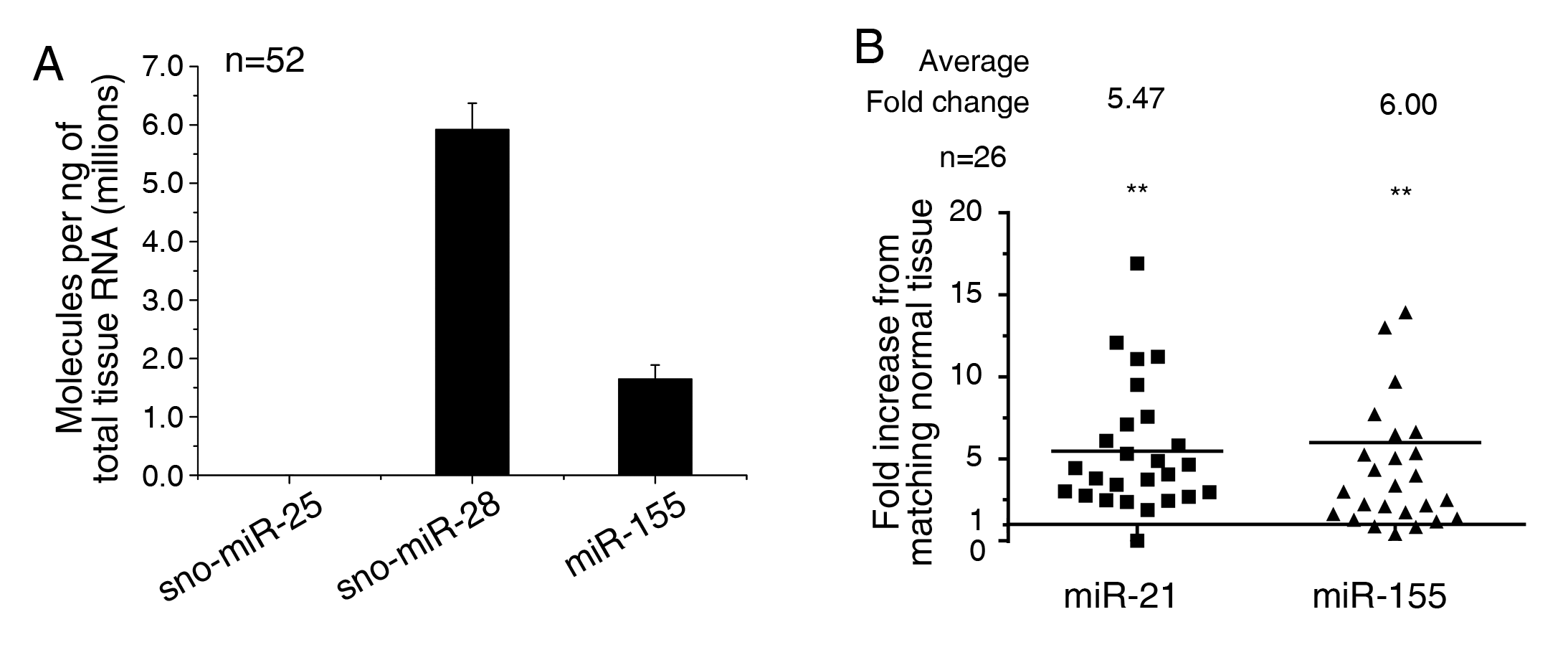

Supplement: S2 Fig — (A) The standard curves in S1 Fig were used to define a standard curve for the absolute expression of miR-155, sno-miR-28 and sno-miR-25 in 26 breast tumour tissues and paired normal adjacent breast tissues using TaqMan assay and RT-PCR. The average expression levels of sno-miR-28, miR-155, and sno-miR-25 are shown in millions of molecules per nanogram (ng) of total RNA. (B) miR-21 and miR-155 expression was determined as positive controls using Taqman assay and RT-PCR in breast tumours compared with paired normal tissue. (TIF) [file pone.0129190.s002.tif]

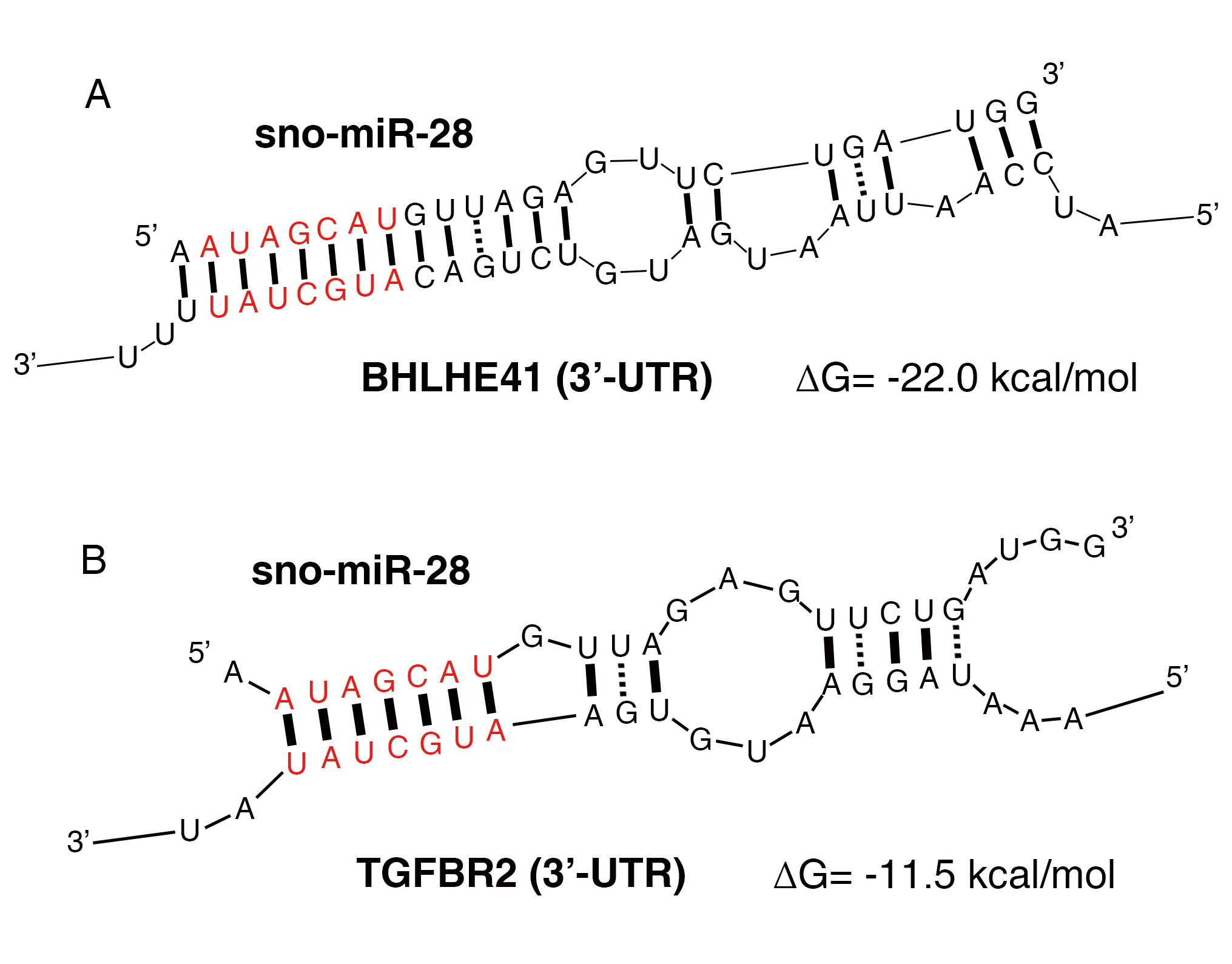

Supplement: S3 Fig — Bioinformatics analysis showed the binding of sno-miR-28 to BHLHE41 and TGFBR2 3’-UTR in a similar manner as other miRNAs binding to their targets. The schematic graphs are made to show the proposed binding sites for sno-miR-28. The seed-recognizing sites are marked in red; hypothesized duplexes formed by the interaction of the binding sites of the 3’-UTR of TAF9B and sno-miR-28 are illustrated, and the predicted free energy of the hybrids were indicated. The solid lines between two chains represent hydrogen bonds between adenine (A)-uracil (U) pairs and guanine (G)-cytosine (C) pairs, whereas dashed lines represent G-U pairings. (TIF) [file pone.0129190.s003.tif]
