# Supplementary material for: p53 Represses the Oncogenic Sno-MiR-28 Derived from a SnoRNA
Source: PLoS One. 2015 Jun 10;10(6):e0129190. doi: 10.1371/journal.pone.0129190 (PMC4465335; doi:10.1371/journal.pone.0129190)
Supplement: S1 Table — As wild-type p53 was induced in H1299 cells, Affymetrix gene expression profiling identified a list of snoRNAs that were repressed. These snoRNAs are listed with their host genes. (PDF) [file pone.0129190.s004.pdf]

Table 1. SNORDs or SNORAs repressed by wild-type p53 in H1299 cells

Gene Name	Accession Number	Fold Repression	Location	Precursor Transcript
SNORD22	NR_000008	2.19	11q13	SNHG1
SNORD25	NR_002565	2.69		
SNORD26	NR_002564	3.12		
SNORD27	NR_002563	2.64		
SNORD28	NR_002562	2.74		
SNORD30	NR_002561	2.11		
SNORD31	NR_002560	1.91		
SNORD74	NR_002579	1.84	1q25.1	SNHG2
SNORD75	NR_003941	1.81		
SNORD78	NR_003944	1.53		
SNORD50A	NR_002743	1.50	6q14.3	SNHG5
SNORD50B	NR_003044	2.09		
SNORD42B	NR_000013	1.87	17q11	RPL23A
SNORD4A	NR_000010	1.63		
SNORD4B	NR_000009	2.16		
SNORA75	NR_002921	2.07	2q37.1	NCL
SNORA61	NR_002987	1.66	1p35.5	SNHG12
SNORD34	NR_000019	1.77	19q13.3	RPL13A
SNORA45	NR_002977	2.07	11p15.4	RPL27A
SNORA64	NR_002326	1.55	16p13.3	RPS2
SNORD73A	NR_000007	1.74	4q31.2	RPS3A
SNORA9	NR_002952	1.68	7p13	C7orf40
SNORA56	NR_002984	1.99	Xq28	DKC1
SNORD96A	NR_002592	1.61	5q35.3	GNB2L1
SNORA71B	NR_002910	1.57	20q11.23	LOC388796
SNORA55	NR_002983	1.67	1p34.3	PABPC4
SNORD45C	NR_003042	1.55	1p31.1	RABGGTB
SNORA40	NR_002973	1.62	11q21	-
SNORD3B-2	NR_003924	2.68	17p11.2	-
SNORD5	NR_003033	1.70	11q21	-
