# Supplementary material for: p53 Represses the Oncogenic Sno-MiR-28 Derived from a SnoRNA
Source: PLoS One. 2015 Jun 10;10(6):e0129190. doi: 10.1371/journal.pone.0129190 (PMC4465335; doi:10.1371/journal.pone.0129190)
Supplement: S2 Table — When wild-type p53 was induced in WE-68 cells, Affymetrix gene expression profiling identified a list of snoRNAs that were repressed. These snoRNAs are shown with their host genes. (PDF) [file pone.0129190.s005.pdf]

Table 2. SNORDs or SNORAs repressed by wild-type p53 upon nutlin-3a treatment

Gene Name	Accession Number	Fold Repression	Location	Precursor Transcript
SNORD22	NR_000008	1.53	11q13	SNHG1
SNORD25	NR_002565	2.09		
SNORD26	NR_002564	1.50		
SNORD27	NR_002563	1.59		
SNORD28	NR_002562	1.51		
SNORA75	NR_002921	1.56	2q37.1	NCL
