# Supplementary material for: p53 Represses the Oncogenic Sno-MiR-28 Derived from a SnoRNA
Source: PLoS One. 2015 Jun 10;10(6):e0129190. doi: 10.1371/journal.pone.0129190 (PMC4465335; doi:10.1371/journal.pone.0129190)
Supplement: S3 Table — Primers used in this study are listed by targets and usage. (DOC) [file pone.0129190.s006.doc]

| **Target gene** | **Forward primer (5’-3’)** | **Reverse Primer (5’-3’)** | **Description** |
| --- | --- | --- | --- |
| **p53** | CCCCTCCTGGCCCCTGTCATCTTC | GCAGCGCCTCACAACCTCCGTCAT | For RT-PCR detection of p53 mRNA |
| **TAF9B** | CCGCGGATGACGAGTGGCTG | TCAGCACGACACTGGATTGCCA | For RT-PCR detection of TAF9B mRNA |
| **SNHG1** | CGCGCACGTTGGAACCGAAG | GCTGGCCCTTTGAGCCAAGCA | For RT-PCR detection of SNHG1 mRNA |
| **SNORD25** | TGATGAGGACCTTTTCACAGACCT | TCAGAGTTATTTATCCTCACGGAGC | For RT-PCR detection of SNORD25 |
| **SNORD28** | AGCTGATGTTCTGTGAGGTACA | GCCATCAGAACTCTAACATGCTATT | For RT-PCR detection of SNORD28 |
| **CDKN1A** | TGGACCTGGAGACTCTCAGGGTCG | TTAGGGCTTCCTCTTGGAGAAGATC | For RT-PCR detection of p21 mRNA |
| **RRM2B** | GCCAGGACTCACTTTTTCCA | TCAGGCAAGCAAAGTCACAG | For RT-PCR detection of p53R2 mRNA |
| **CCNG1** | GTCCCATTGGCAACTGACTT | TGACATGCCTTCAGTTGAGC | For RT-PCR detection of CCNG1 mRNA |
| **FAS** | ATGCTGGGCATCTGGACCCT | GCCATGTCCTTCATCACACAA | For RT-PCR detection of FAS mRNA |
| **HDM2** | TCTACAGGGACGCCATCGA | CTGATCCAACCAATCACCTGAA | For RT-PCR detection of HDM2 mRNA |
| **ChIP negative site** | GCTGTGGTGTGTAGGAAGCAGCAG | AAAGGAGGCCAAAGTCGGGCAATG | Negative control for p53 ChIP |
| **SNHG1 p53-RE** | AGACAATGGAGAGGCACTGAACTGA | GGTCAGGTCCCACTTCAGCTCACAA | For ChIP PCR of p53-RE in SNHG1 promoter |
| **CDKN1A ChIP** | GTGGCTCTGATTGGCTTTCTG | CTGAAAACAGGCAGCCCAAG | CDKN1A ChIP primers  For cloning of TAF9B 3'-UTR into the psiCHECK2 vector |
| **TAF9B 3'-UTR** | GCAGCTAGCAAGGAAAGTTGGTTTTGA | GCTGCGGCCGCTATTAACCTTGGAACAAA |
| **TAF9B-mut-1** | TGATAACTCTTCCATGTTTCACATC | GCAATAATTCCTTACAGTCATTTTTTTTTTTTTTTTTTGAATTCAATTTTCACATATACCAATT | For introducing a mutation at the sno-miR-28 recognition sequence in the TAF9B 3'-UTR cloned in the psiCHECK2 vector |
| **TAF9B-mut-2** | AATTGGTATATGTGAAAATTGAATTCAAAAAAAAAAAAAAAAAATGACTGTAAGGAATTATTGC | CAAACCCTAACCACCGCTTA |  |
